# Standardised inventories of spiders (Arachnida, Araneae) on touristic trails of the native forests of the Azores (Portugal)

**DOI:** 10.3897/BDJ.9.e62886

**Published:** 2021-04-16

**Authors:** Rui Carvalho, Pedro Cardoso, Artur Gil, Maria Teresa Ferreira, Cândida Ramos, Lucas Lamelas-Lopez, Fernando Pereira, Jagoba Malumbres-Olarte, Alejandra Ros-Prieto, Mário Boieiro, Paulo A. V. Borges

**Affiliations:** 1 cE3c – Centre for Ecology, Evolution and Environmental Changes / Azorean Biodiversity Group and Universidade dos Açores, Rua Capitão João d’Ávila, São Pedro, 9700-042, Angra do Heroísmo, Portugal cE3c – Centre for Ecology, Evolution and Environmental Changes / Azorean Biodiversity Group and Universidade dos Açores, Rua Capitão João d’Ávila, São Pedro, 9700-042 Angra do Heroísmo Portugal; 2 Laboratory for Integrative Biodiversity Research (LIBRe), Finnish Museum of Natural History, University of Helsinki, Helsinki, Finland Laboratory for Integrative Biodiversity Research (LIBRe), Finnish Museum of Natural History, University of Helsinki Helsinki Finland; 3 cE3c – Centre for Ecology, Evolution and Environmental Changes / Azorean Biodiversity Group and Universidade dos Açores, Ponta Delgada, Portugal cE3c – Centre for Ecology, Evolution and Environmental Changes / Azorean Biodiversity Group and Universidade dos Açores Ponta Delgada Portugal; 4 IUCN SSC Mid-Atlantic Islands Specialist Group, Angra do Heroísmo, Portugal IUCN SSC Mid-Atlantic Islands Specialist Group Angra do Heroísmo Portugal

**Keywords:** Arthropoda, hiking, recreation ecology, Macaronesia, endemic species, checklist

## Abstract

**Background:**

The sharp increase in tourist visitation of the Azores Archipelago from 2015 onwards raised concerns about the impacts of recreational tourism on native habitats. In response, a project was financed by the Azorean Government to investigate the drivers of biodiversity erosion associated with recreational tourism. Here, we present the data on spider biodiversity found on trails located within the native Azorean forests as they are home to several endemic species of great conservation value. We applied an optimised and standardised sampling protocol (COBRA) in twenty-three plots located in five trails on Terceira and São Miguel Islands and assessed diversity and abundance of spider species at different distances from the trail head and the trail itself.

**New information:**

Of the 45 species (12435 specimens) collected, 13 were endemic to the Azores (9690 specimens), 10 native non-endemic (2047 specimens) and 22 introduced (698 specimens). This database will be the baseline of a long-term monitoring project for the assessment of touristic impacts on native forest trails. This methodology can also be used on other habitats and biogeograhical regions.

## Introduction

In the Azores, as in many other temperate, semi-tropical and tropical islands, historical patterns of habitat loss have typically resulted in lowland clearance, meaning that the last remnants of the pre-human pristine forest that covered the major parts of oceanic islands are in the mountain areas (Gaspar et al. 2011). The communities of these mountain forests are of critical importance for the protection of current island biodiversity since they are home to many Azorean endemic species (Borges et al. 2017, Borges et al. 2018, Malumbres-Olarte et al. 2019) and provide a variety of ecosystem services (e.g. water storage, erosion control, pollination, pest-control, food supply, recreation and tourism), contributing to the local economy and welfare (Fernandez-Palacios et al. 2017).

The recent increase in recreational tourist activities in native habitats of the Azores (SREA 2018) raises concerns about the use of trails being a threat to the already imperilled native forest biodiversity. Hiking trails in particular have been found to be promoting the spread of invasive plants (Barros and Pickering 2014), which may cause adverse cascading effects on arthropods.

The spider communities of the Azores are exceptionally well known due to ongoing inventorying and monitoring projects carried out since 1999 (Borges et al. 2016, Emerson et al. 2017, Malumbres-Olarte et al. 2019). The protocol used in NETBIOME ISLANDBIODIV and in this project is part of a long term monitoring proposal for oceanic islands (Borges et al. 2018).

## General description

### Purpose

We aimed to characterise the richness and abundance of spiders in areas surrounding trails in native Azorean forest and to assess if the distance to the head of hiking trails or to the trail itself explains shifts in spider community composition, compared with areas undisturbed by tourists.

## Project description

### Title

Spiders (Arachnida, Araneae) from Azorean native forest trails

### Personnel

Rui Carvalho led the sampling in the field with the participation of Alejandra Ros-Prieto, Cândida Ramos, Fernando Pereira, Jagoba Malumbres-Olarte, Maria T. Ferreira, Mário Boieiro, Lucas Lamelas-López and Paulo A. V. Borges.

### Study area description

**Figure 1.**
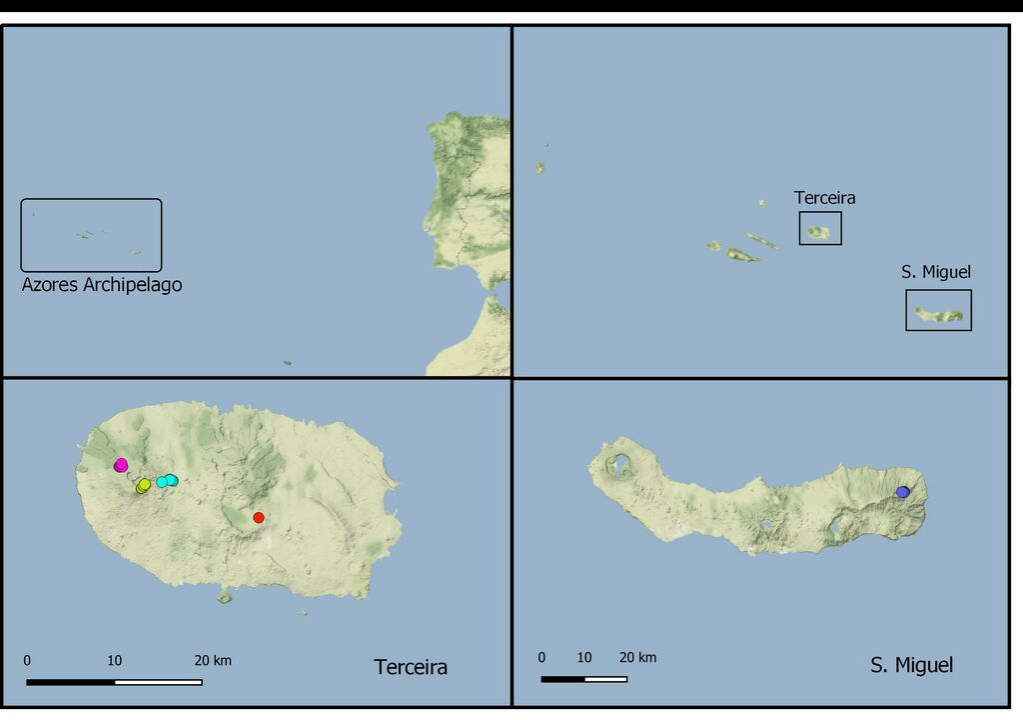
Location of trails in Terceira: Lagoinha (pink), Santa Bárbara (yellow), Mistérios Negros (blue), Guilherme Moniz (red); in S. Miguel: Malhadas (purple).

We focused on the Azorean forests of Terceira and São Miguel Islands, as they have pedestrian trails going through native forests with a relevant level of visitation (Fig. 1). Terceira Island (area: 400.6 km²; elevation: 1021.14 m) and São Miguel Island (area: 744.6 km²; elevation: 1103 m) are two of the nine islands from the Azores Archipelago. The climate in the Azores is temperate oceanic, with regular and abundant rainfall, with high levels of relative humidity and persistent winds, mainly during winter and autumn seasons. Terceira Island is known for the presence of some very important pristine areas at high elevation (Gaspar et al. 2011).

### Funding

This research was supported by the Rui Carvalho Ph.D. DRCT scholarship from the Azores Government (M3.1.a/F/135/2015). Data was obtained mostly during the Ph.D. DRCT scholarship, but some samples ("Control 250", see below) are from a previous project (ERA-Net NetBiome research framework, financed through Portuguese FCT-NETBIOME ISLANDBIODIV grant 0003/2011).

## Sampling methods

### Study extent

We selected six 50 x 50 m sampling sites in native forest patches along the studied trails, at increasing distances from the trail head: 0 m, 50 m and 250 m. Another sampling site, termed Max, was set independently from distance - it was located where the forest adjacent to the trail was most pristine. Finally, two control sites were selected at 50 m and 250 m from the closest trail point (Table 1). This setup is repeated at each trail. In Terceira, the Control 250 data were retrieved from NETBIOME-ISLANDBIODIV samples from 2012 (see Malumbres-Olarte et al. 2019).

### Sampling description

The inventory COBRA (Conservation Oriented Biodiversity Rapid Assessment) protocol (Cardoso 2009) was used at the most pristine area in the studied fragment, firstly to assess whether completeness is sufficient to use the less time-intensive protocols; secondly, in order to be used as alpha and beta diversity baselines (Borges et al. 2018). It is composed of four hours of aerial search, four hours of tree beating, four hours of vegetation sweeping and pitfall sampling using 48 traps. The traps containing propylene glycol were active for 15 days and, during sample collection, they were arranged in groups of four to make 12 sample units. For the remaining sampling areas of each trail, the much less intensive monitoring COBRA protocol was used. It is composed of four hours of aerial search and two hours of beating trees using a drop cloth (see Borges et al. 2018 for details). The COBRA protocol has been proposed as part of standard inventorying and monitoring programmes targeting spiders and beetles and has been used on island and continental ecosystems, from subarctic regions to the tropics (Cardoso 2009, Borges et al. 2018, Malumbres-Olarte et al. 2019, Malumbres-Olarte et al. 2020).

### Quality control

All the spider specimens were first sorted into morphospecies by R Carvalho and later identified by a trained taxonomist (one of the authors: PAV Borges).

## Geographic coverage

### Description

Terceira and São Miguel Islands, Azores, Portugal

**Bounding Coordinates**: South West [37.579, -27.466], North East [39.045, -25.049]

## Taxonomic coverage

### Taxa included

**Table:** 

Rank	Scientific Name	Common Name
order	Araneae	Spiders

## Traits coverage

Macías-Hernández et al. (2020) published the database of functional traits for all species in the study.

## Temporal coverage

### Notes

July to August 2012 for the Control 250 samples; July to October 2017 for all other samples.

## Collection data

### Collection name

Dalberto Teixeira Pombo insect collection at the University of Azores.

### Collection identifier

DTP

### Specimen preservation method

All specimens were preserved in 96% ethanol.

### Curatorial unit

Dalberto Teixeira Pombo insect collection at the University of the Azores (Curator: Paulo A. V. Borges)

## Usage licence

### Usage licence

Open Data Commons Attribution License

### IP rights notes

CC-BY 4.0

## Data resources

### Data package title

Diversity of Spiders from Azorean Trails

### Resource link

https://www.gbif.org/dataset/76e75816-b0dc-4460-9de2-294f3e05ad83

### Alternative identifiers

https://doi.org/10.15468/wgnw57

### Number of data sets

1

### Data set 1.

#### Data set name

Diversity of Spiders from Azorean Trails

#### Data format

Darwin Core Archive

#### Number of columns

59

#### Download URL

http://ipt.gbif.pt/ipt/resource?r=spiders_of_azorean_trails

#### Data format version

version 1

#### Description

The following data table includes all the records for which a taxonomic identification of the species was possible. The dataset submitted to GBIF (Global Biodiversity Information Facility) is structured as a sample event dataset, with two tables: event (as core) and occurrences. The data in this sampling event resource have been published as a Darwin Core Archive (DwCA), which is a standardised format for sharing biodiversity data as a set of one or more data tables. The core data file contains 194 records (eventID) and the occurrences file 1290 records (occurrenceID). This IPT (integrated publishing toolkit) archives the data and thus serves as the data repository. The data and resource metadata are available for download from Carvalho et al. (2021).

**Data set 1.** 

Column label	Column description
Table of Sampling Events	Table with sampling events data (beginning of table)
id	Unique identification code for sampling event data
eventID	Identifier of the events, unique for the dataset
stateProvince	Name of the region of the sampling site
islandGroup	Name of archipelago
island	Name of the island
country	Country of the sampling site
countryCode	ISO code of the country of the sampling site
municipality	Municipality of the sampling site
decimalLongitude	Approximate centre point decimal longitude of the field site in GPS coordinates
decimalLatitude	Approximate centre point decimal latitude of the field site in GPS coordinates
geodeticDatum	The ellipsoid, geodetic datum or spatial reference system (SRS) upon which the geographic coordinates given in decimalLatitude and decimalLongitude are based
coordinateUncertaintyInMetres	Uncertainty of the coordinates of the centre of the sampling plot
coordinatePrecision	Precision of the coordinates
georeferenceSources	A list (concatenated and separated) of maps, gazetteers or other resources used to georeference the Location, described specifically enough to allow anyone in the future to use the same resources.
locationID	Identifier of the location
locationRemarks	Details on the locality site
locality	Name of the locality
minimumElevationInMetres	The lower limit of the range of elevation (altitude, usually above sea level), in metres.
maximumElevationInMetres	The upper limit of the range of elevation (altitude, usually above sea level), in metres.
habitat	The surveyed habitat
year	Year of the event
month	Month of the event
day	Day of the event
eventRemarks	Comments or notes about the Event
samplingProtocol	The sampling protocol used to capture the species
sampleSizeValue	The numeric amount of time spent in each sampling
sampleSizeUnit	The unit of the sample size value
samplingEffort	The amount of time of each sampling
fieldNumber	An identifier given to the event in the field. Often serves as a link between field notes and the Event.
eventDate	Date or date range the record was collected
Table of Species Occurrence	Table with species abundance data (beginning of new table)
id	Unique identification code for species abundance data
type	Type of the record, as defined by the Public Core standard
licence	Reference to the licence under which the record is published
institutionID	The identity of the institution publishing the data
collectionID	The identity of the collection publishing the data
institutionCode	The code of the institution publishing the data
collectionCode	The code of the collection where the specimens are conserved
datasetName	Name of the dataset
basisOfRecord	The nature of the data record
dynamicProperties	The name of the scientific project funding the sampling
occurrenceID	Identifier of the record, coded as a global unique identifier
recordedBy	Name of the person who performed the sampling of the specimens
individualCount	Total number of individuals captured
sex	The sex and quantity of the individuals captured
lifeStage	The life stage of the organisms captured
establishmentMeans	The process of establishment of the species in the location, using a controlled vocabulary: 'naturalised', 'introduced', 'endemic', "unknown"
eventID	Identifier of the events, unique for the dataset
scientificName	Complete scientific name including author and year
kingdom	Kingdom name
phylum	Phylum name
class	Class name
order	Order name
family	Family name
genus	Genus name
specificEpithet	Specific epithet
taxonRank	Lowest taxonomic rank of the record
scientificNameAuthorship	Name of the author of the lowest taxon rank included in the record

## Additional information

### Results

We collected a total of 12435 specimens belonging to 45 species of spiders. A total of 13 species are endemic to the Azores Archipelago (9690 specimens), 10 are native non-endemic (2047 specimens) and 22 are introduced (698 specimens) (Table 2, Table 3).

The five most abundant species were *Gibbaranea occidentalis* Wunderlich, 1989 (3635 specimens) (endemic), *Sancus acoreensis* (Wunderlich, 1992) (3096 specimens) (endemic), *Savigniorrhipis acoreensis* Wunderlich, 1992 (1595 specimens) (endemic), *Lathys dentichelis* (Simon, 1883) (1361 specimens) (native non-endemic) and *Rugathodes acoreensis* Wunderlich, 1992 (799 specimens) (endemic). These five species accounted for 84% of all individuals of the total. The most abundant introduced species was *Metellina merianae* Scopoli, 1763 with 279 specimens.

This database will be used in future studies where the variation of the spider communites amongst the various sites will be tested against variables that are known to be relevant for understanding the impact of touristic activities, such as the distance to the trail head and the distance from the sampling area to the nearest trail point. We will use GLMMs, where the trail identity will be used as random effect and the edge effect will be added as an independent variable in order to avoid spurious results. This will respond to the questions of whether there is a detectable effect of recreational activities on the spiders community structure and what is contributing to this ecological shift.

Contrary to Canary Islands and Madeira, the Azorean Archipelago has not yet experienced continuous high levels of visitation. This sampling was made at the early times of a noticeably higher touristic pressure in the Azores and will allow for future monitoring events to have a comparable baseline and better isolate the touristic factors from others, thus improving the management outlook on tourism's ecological effects on spider communities.

## Figures and Tables

**Table 1.** Island, fragment, trail name and coordinates of sampling sites

Island	Fragment	Trail	Sampling sites	Latitude	Longitude
Terceira	Santa Bárbara	Lagoinha	0	38.7496	-27.3340
Terceira	Santa Bárbara	Lagoinha	50	38.74946	-27.3333
Terceira	Santa Bárbara	Lagoinha	250	38.7497	-27.3320
Terceira	Santa Bárbara	Lagoinha	Control 50	38.7496	-27.3304
Terceira	Santa Bárbara	Lagoinha	Control 250	38.7521	-27.3313
Terceira	Santa Bárbara	Mistérios Negros	0	38.7383	-27.2786
Terceira	Santa Bárbara	Mistérios Negros	50	38.7383	-27.2789
Terceira	Santa Bárbara	Mistérios Negros	250	38.7390	-27.2801
Terceira	Santa Bárbara	Mistérios Negros	Max	38.7394	-27.2824
Terceira	Santa Bárbara	Mistérios Negros	Control 50	38.7390	-27.2819
Terceira	Santa Bárbara	Mistérios Negros	Control 250	38.7372	-27.2899
Terceira	Santa Bárbara	Santa Bárbara	0	38.7322	-27.3111
Terceira	Santa Bárbara	Santa Bárbara	50	38.7325	-27.3106
Terceira	Santa Bárbara	Santa Bárbara	250	38.7336	-27.3088
Terceira	Santa Bárbara	Santa Bárbara	Max	38.7347	-27.3073
Terceira	Santa Bárbara	Santa Bárbara	Control 50	38.7348	-27.3090
Terceira	Santa Bárbara	Santa Bárbara	Control 250	38.7356	-27.3074
Terceira	Guilherme Moniz	Guilherme Moniz	0	38.7087	-27.1904
São Miguel	Pico da Vara	Malhadas	0	37.8170	-25.1848
São Miguel	Pico da Vara	Malhadas	50	37.8164	-25.1855
São Miguel	Pico da Vara	Malhadas	250	37.8157	-25.1864
São Miguel	Pico da Vara	Malhadas	Max	37.8163	-25.1900

**Table 2.** Diversity and abundance for the collected species, according to biogeographic origin and sampling area.

	Endemic		Native		Introduced	
Trail / Sampling Area	Species richness	Abundance	Species richness	Abundance	Species richness	Abundance
Lagoinha 0	5	479	10	62	5	7
Lagoinha 50	7	364	14	91	7	15
Lagoinha 250	5	534	12	64	7	11
Lagoinha Control 50	9	419	15	174	3	5
Lagoinha Control 250	5	417	14	187	10	27
Mistérios Negros 0	6	466	17	94	14	33
Mistérios Negros 50	8	421	15	55	7	14
Mistérios Negros 250	8	418	18	119	18	184
Mistérios Negros Max	9	628	20	64	15	57
Mistérios Negros Control 50	6	993	13	187	10	27
Mistérios Negros Control 250	9	394	17	128	7	12
Santa Bárbara 0	8	325	13	17	2	12
Santa Bárbara 50	7	230	13	38	5	22
Santa Bárbara 250	6	417	11	43	3	7
Santa Bárbara Max	8	410	15	40	4	6
Santa Bárbara Control 50	7	463	16	26	6	10
Santa Bárbara Control 250	10	405	17	75	8	17
Guilherme Moniz 0	8	903	26	220	20	57
Malhadas 0	6	148	18	41	13	20
Malhadas 50	7	245	22	63	19	79
Malhadas 250	5	232	14	43	10	30
Malhadas Max	7	378	22	217	35	46

**Table 3.** Spider species abundance in each study area and their biogeographic origin. Abbreviations: Biogeographic origin (Biog. origin); Endemic (END); Introduced (INT); Native (NAT).

Family	Scientific name	Biog. origin	Lagoinha	Mistérios Negros	Santa Bárbara	Guilherme Moniz	Malhadas
Araneidae	*Gibbaranea occidentalis* Wunderlich, 1989	END	813	1754	365	563	140
Araneidae	*Mangora acalypha* (Walckenaer, 1802)	INT	0	138	0	1	18
Araneidae	*Neoscona crucifera* (Lucas, 1838)	INT	0	0	0	0	3
Clubionidae	*Porrhoclubiona decora* (Blackwall, 1859)	NAT	2	1	0	4	9
Dictynidae	*Lathys dentichelis* (Simon, 1883)	NAT	337	507	151	168	198
Dictynidae	*Nigma puella* (Simon, 1870)	INT	0	0	0	0	1
Dysderidae	*Dysdera crocata* C. L. Koch, 1838	INT	0	3	0	1	0
Cheiracanthiidae	*Cheiracanthium erraticum* (Walckenaer, 1802)	INT	51	7	20	0	27
Linyphiidae	*Acorigone acoreensis* (Wunderlich, 1992)	END	5	4	11	1	0
Linyphiidae	*Agyneta decora* (O.P.-Cambridge, 1871)	INT	0	0	3	0	2
Linyphiidae	*Canariphantes acoreensis* (Wunderlich, 1992)	END	12	17	91	0	0
Linyphiidae	*Erigone atra* Blackwall, 1833	INT	1	0	0	0	1
Linyphiidae	*Erigone autumnalis* Emerton, 1882	INT	0	1	0	1	1
Linyphiidae	*Erigone dentipalpis* (Wider, 1834)	INT	0	0	0	0	1
Linyphiidae	*Meioneta fuscipalpa* (C.L. Koch, 1836)	INT	0	0	0	1	0
Linyphiidae	*Mermessus bryantae* (Ivie & Barrows, 1935)	INT	0	1	0	0	0
Linyphiidae	*Mermessus fradeorum* (Berland, 1932)	INT	0	0	0	2	0
Linyphiidae	*Microlinyphia johnsoni* (Blackwall, 1859)	NAT	16	19	12	16	0
Linyphiidae	*Minicia floresensis* Wunderlich, 1992	END	1	0	3	0	7
Linyphiidae	*Neriene clathrata* (Sundevall, 1830)	INT	0	0	0	0	2
Linyphiidae	*Oedothorax fuscus* (Blackwall, 1834)	INT	0	0	0	1	1
Linyphiidae	*Palliduphantes schmitzi* (Kulczynski, 1899)	NAT	2	0	3	0	0
Linyphiidae	*Porrhomma borgesi* Wunderlich, 2008	END	0	1	1	0	0
Linyphiidae	*Prinerigone vagans* (Audouin, 1826)	INT	0	0	0	1	4
Linyphiidae	*Savigniorrhipis acoreensis* Wunderlich, 1992	END	430	537	384	60	184
Linyphiidae	*Tenuiphantes miguelensis* (Wunderlich, 1992)	NAT	97	22	20	0	46
Linyphiidae	*Tenuiphantes tenuis* (Blackwall, 1852)	INT	3	2	2	1	14
Linyphiidae	*Walckenaeria grandis* (Wunderlich, 1992)	END	1	0	11	0	0
Lycosidae	*Pardosa acorensis* Simon, 1883	END	3	4	45	0	72
Mimetidae	*Ero furcata* (Villers, 1789)	INT	4	23	0	2	10
Pisauridae	*Pisaura acoreensis* Wunderlich, 1992	END	3	13	57	8	21
Salticidae	*Macaroeris cata* (Blackwall, 1867)	NAT	97	55	16	28	63
Salticidae	*Macaroeris diligens* (Blackwall, 1867)	NAT	0	0	1	0	0
Salticidae	*Neon acoreensis* Wunderlich, 2008	END	0	2	0	1	0
Tetragnathidae	*Metellina merianae* (Scopoli, 1763)	INT	5	146	15	35	78
Tetragnathidae	*Sancus acoreensis* (Wunderlich, 1992)	END	831	621	974	165	505
Theridiidae	*Cryptachaea blattea* (Urquhart, 1886)	INT	0	0	1	1	1
Theridiidae	*Lasaeola oceanica* Simon, 1883	END	53	105	4	2	6
Theridiidae	*Rhomphaea nasica* (Simon, 1873)	INT	0	0	0	1	0
Theridiidae	*Rugathodes acoreensis* Wunderlich, 1992	END	61	263	304	103	68
Theridiidae	*Steatoda nobilis* (Thorell, 1875)	INT	1	6	1	9	11
Theridiidae	*Theridion musivivum* Schmidt, 1956	NAT	0	0	0	0	2
Thomisidae	*Xysticus cor* Canestrini, 1873	NAT	27	42	36	4	46
Thomisidae	*Xysticus nubilus* Simon, 1875	INT	0	0	32	0	0
